# Complexes of Ir^III^-Octaethylporphyrin with Peptides as Probes for Sensing Cellular O_2_

**DOI:** 10.1002/cbic.201200083

**Published:** 2012-04-24

**Authors:** Klaus Koren, Ruslan I Dmitriev, Sergey M Borisov, Dmitri B Papkovsky, Ingo Klimant

**Affiliations:** [a] Institute of Analytical Chemistry and Food Chemistry, Graz University of TechnologyStremayrgasse 9, 8010 Graz (Austria); [b] Laboratory of Biophysics and Bioanalysis, Department of Biochemistry, University College CorkCork (Ireland)

**Keywords:** cell-penetrating peptide, cellular oxygen sensing, histidine ligands, Ir–porphyrin complexes, phosphorescence quenching

## Abstract

Ir^III^–porphyrins are a relatively new group of phosphorescent dyes that have potential for oxygen sensing and labeling of biomolecules. The requirement of two axial ligands for the Ir^III^ ion permits simple linkage of biomolecules by a one-step ligand-exchange reaction, for example, using precursor carbonyl chloride complexes and peptides containing histidine residue(s). Using this approach, we produced three complexes of Ir^III^–octaethylporphyrin with cell-penetrating (Ir1 and Ir2) and tumor-targeting (Ir3) peptides and studied their photophysical properties. All of the complexes were stable and possessed bright, long-decay (unquenched lifetimes exceeding 45 μs) phosphorescence at around 650 nm, with moderate sensitivity to oxygen. The Ir1 and Ir2 complexes showed positive staining of a number of mammalian cell types, thus demonstrating localization similar to endoplasmic reticulum and ATP- and temperature-independent intracellular accumulation (direct translocation mechanism). Their low photo- and cytotoxicity allows intracellular oxygen to be probed.

## Introduction

Molecular oxygen (O_2_) is one of the key metabolites and functional parameters of live cells and tissues that reflects their respiration activity, mitochondrial function, and oxygenation state.^1^, ^2^ Numerous methods for direct and indirect assessment of O_2_ in the cell and tissue were proposed, which include Clark (micro)electrodes,^3^, ^4^ electron paramagnetic resonance (EPR),^5^, ^6^ optical sensing,^4^, ^7^, ^8^ and special “hypoxia” probes (HIF constructs, nitroimidazoles, etc.).^9^ In recent years, new methods for minimally invasive sensing of intracellular O_2_ (icO_2_) were introduced which use nitroxyl and esterified trityl (triarylmethyl) radicals,^5^, ^6^, ^10^, ^11^ O_2_-sensitive genetically encoded GFP constructs,^12^ endogenous mitochondrial protoporphyrin IX,^13^, ^14^ Ir-BTP, Ru–polypirydyl, or Pt– and Pd–porphyrin probes based on cell-penetrating peptide(s) or nanoparticles.^15^–^27^

Planar Pt^II^ and Pd^II^ complexes of porphyrin dyes exhibit strong phosphorescence at room temperature which is readily quenched by O_2_.^28^ Characteristic spectral properties of such compounds allow their use in different O_2_-sensing materials and detection modalities, including time-resolved fluorescence/phosphorescence,^29^ phosphorescence quenching microscopy/FLIM,^30^–^32^ or ratiometric detection.^22^, ^33^ With the development of cell-targeting vectors^34^–^36^ and nanoparticle technology,^37^, ^38^ intracellular delivery of such sensors has become possible. Thus, PtCP conjugates with oligoarginine or bactenecin 7 peptides, or PtTFPP and PtOEP dyes embedded in positively charged nanoparticles (with or without additional cell-penetrating coating) were found useful for biological and physiological studies.^17^–^21^, ^24^ At the same time, mechanisms of their transport into the cell and control of intracellular localization and fate remain poorly understood and require further investigation of structure–activity relationships.

Ir^III^–porphyrins represent a relatively new group of phosphorescent dyes^39^ which have not been explored in detail to date. The six-coordination geometry of the Ir^III^ central atom makes these dyes attractive for synthesis of new supramolecular structures. The tetrapyrrole macrocycle occupies four coordination sites, while the remaining two axial sites can be used to introduce nitrogen-containing heterocycles such as pyridine and imidazole.^39^ This can be used to design new indicator dyes and supramolecular structures with attractive features and spectral properties similar to those of Pt–porphyrins.

In this study, we further develop this synthetic approach by attaching short peptide sequences to the Ir–octaethylporphyrin (Ir–OEP) via histidine residues (structural analogues of imidazoles). These complexes were then characterized spectroscopically and tested on a number of different cell lines with the aim of producing new phosphorescent probes with cell permeating and/or binding capabilities.

## Results and Discussion

### Synthesis of the conjugates

**Figure 1:**
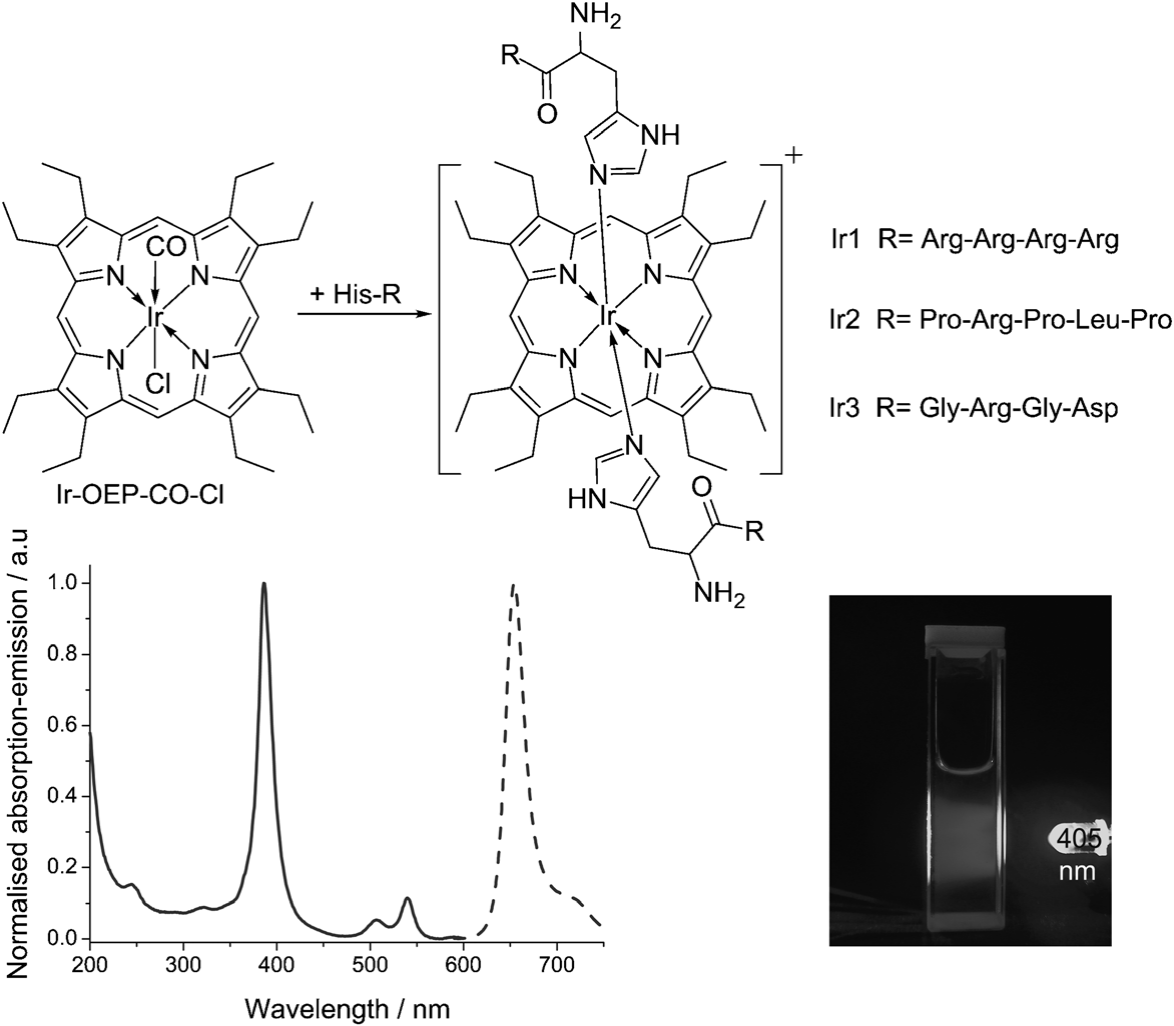
Structures and electronic spectra of the new Ir–OEP complexes. Starting from Ir–OEP–CO-Cl, the complexes were obtained by treatment with histidine-containing peptides (top). Absorption and emission spectra of Ir1and a photographic image of the bright red emission in deoxygenated solution under 405 nm LED excitation.

Previously, several complexes with nitrogen-containing heterocycles were prepared by using Ir–OEP–CO-Cl as a precursor dye and simple ligand-exchange reactions (Figure 1) in 2-ethoxyethanol, which at elevated temperature dissolves both the hydrophobic dye and polar ligands (e.g., carboxyimidazole).^39^ A similar strategy was applied to couple the Ir–OEP–CO-Cl with short peptide sequences via their histidine residues. This method also allows for synthesis of mono- and hetero-substituted Ir^III^–porphyrins; however, in this study, we mainly focused on symmetric disubstituted Ir–OEP derivatives (Table 1).

**Table 1:** Photophysical properties for the synthesized conjugates

Conjugate	*M*_W_ [g mol^−1^]	Charge	*λ*_abs max_ [nm]	*λ*_em max_ [nm]	Q.Y.	*τ*_0_ [μs]
Ir1	2282.8	+9	386, 506, 540	654	0.13,[a] 0.16[b]	58
Ir2	2154.7	+3	388, 508, 539	652	0.08,[a] 010[b]	69
Ir3	1804.1	+1	386, 507, 540	654	0.13,[a] 0.15[b]	47

[a] In PBS.

[b] In PBS with 10 % FBS.

Based on our recent studies with peptide conjugates of coproporphyrin dyes (PtCP, PdCP, CPK),^17^, ^18^ we decided to prepare Ir–OEP complexes with two peptide structures which were expected to provide cell-penetrating ability for the resulting complexes: 1) histidine-tetraarginine, HR_4_ (Ir1), and 2) a truncated fragment of cell-penetrating bactenecin 7 peptide, PRPLP (Ir2). In addition, a complex containing an RGD sequence (known for its ability to bind to tumor cell membranes^40^) was prepared (Ir3). Structures of these complexes are presented in Figure 1. Notably, all of the peptides were amidated at the C terminus in order to retain the positive charge of the complexes and were soluble in 2-ethoxyethanol.

The ligand-exchange reaction performed at elevated temperature produced a characteristic hypsochromic shift in the porphyrin absorption spectrum,^39^ thus indicating formation of the complexes, which were then purified by RP-HPLC. Confirmation of purity and molecular structure of the complexes by ^1^H NMR, HPLC, and MS can be found in Figures S1 and S2 in the Supporting Information. After purification, the conjugates were stored in DMSO or water in the dark at 4 °C and were stable for several months. Conversely, when a peptide without a histidine residue (R_4_) was used, no changes in the absorption of Ir—OEP–CO-Cl and no complex formation were observed, even after prolonged 48 h incubation (results not shown).

### Spectral properties and O_2_ sensitivity

Absorption and emission spectra of the three peptide conjugates were found to be similar (Figure 1). High resemblance to Pt–porphyrins can be seen,^18^ with the Soret band at around 386 nm, Q-bands at 506 and 540 nm, and peptide absorption in the UV region. The conjugates were readily soluble in aqueous solutions; their phosphorescence was not affected by the common media, serum, and additives used in cell culture (Table 1), and quantum yields in deoxygenated solutions ranged from 8 to 16 %, which is comparable to Pt–porphyrins.^28^ Red, long-decay emission of these complexes allows for their sensitive detection by time-resolved fluorimetry (Figure S3). Thus, on a standard microplate TR-F reader (Victor 2), the conjugate Ir1 was detectable at concentrations as low as 1 nm in both deoxygenated and oxygenated solutions (Figure S4). Quenched lifetimes for the complexes in air-saturated solutions were at the lower end of instrument time resolution (limited by Xe flash lamp with a pulse duration of about 10 μs). However, reliable sensing of O_2_ on such instruments by using the rapid lifetime determination (RLD) method^41^ was still possible over the O_2_ range 0–250 μm (0–21 % atmospheric O_2_) (Figure S3 B). Interestingly, Ir2 showed the longest decay time (69 μs) but the lowest quantum yield.

The stability of Ir1 was assessed by exposing it to a competing ligand in aqueous solution. When Ir1 was incubated in PBS with a tenfold molar excess of free histidine for 24 h at 37 °C, no changes on the HPLC chromatogram (no additional peaks) were seen (not shown). This proves that under physiological conditions, dissociation or substitution of the peptide ligands in such complexes is insignificant.

### Cell staining properties of the Ir conjugates

**Figure 2:**
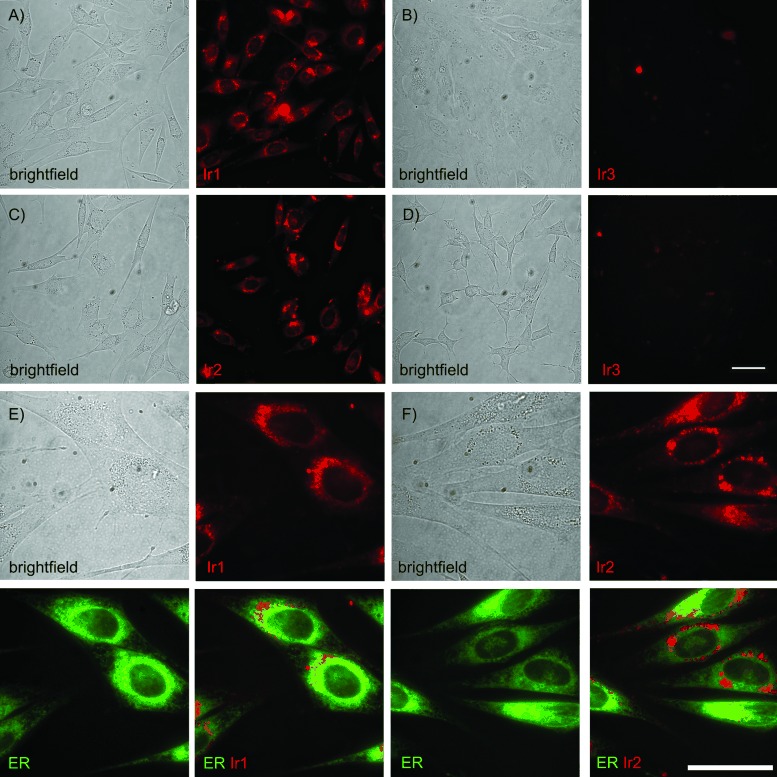
Staining of cells with Ir1, Ir2, and Ir3 probes. A), C), E), and F) brightfield and fluorescent images of MEF, B) HeLa, and D) SH-SY5Y cells. Ir–OEP phosphorescence (red) was recorded by using a 390 nm excitation and 650 nm emission filter set, and ER Tracker Green fluorescence (green) using 490 nm excitation and 530 nm emission filters. Scale bars=50 μm.

The cell-penetrating ability of Ir1 and Ir2 probes was tested using MEF cells as a model. At concentrations of 1–10 μm and incubation times from 6–16 h, both probes demonstrated efficient staining of the cells, accumulating in perinuclear regions and partially colocalizing with the marker for endoplasmic reticulum, ER Tracker Green (Figure 2). Such intracellular localization differs from that of similar peptides conjugated to PtCP dye via its peripheral propionic acid residues.^17^–^19^ Notably, Ir2 demonstrated a small degree of aggregation on the cell surface.

Conjugates Ir1 and Ir2 were further tested in COS-7, HeLa, SH-SY5Y, and PC12 cell lines and with mixed cultures of primary neurons and astrocytes. Positive cytoplasmic staining was observed for all of these cell lines (Figure S5). Such cell specificity of Ir1 and Ir2 probes is significantly higher than for the other O_2_ probes, for which staining of SH-SY5Y and primary neuronal cells was low (RID, unpublished data). This also suggests that Ir1 and Ir2 employ different mechanisms of endocytosis or, perhaps, direct translocation through the plasma membrane (temperature- and ATP-independent^35^, ^42^–^44^).

**Figure 3:**
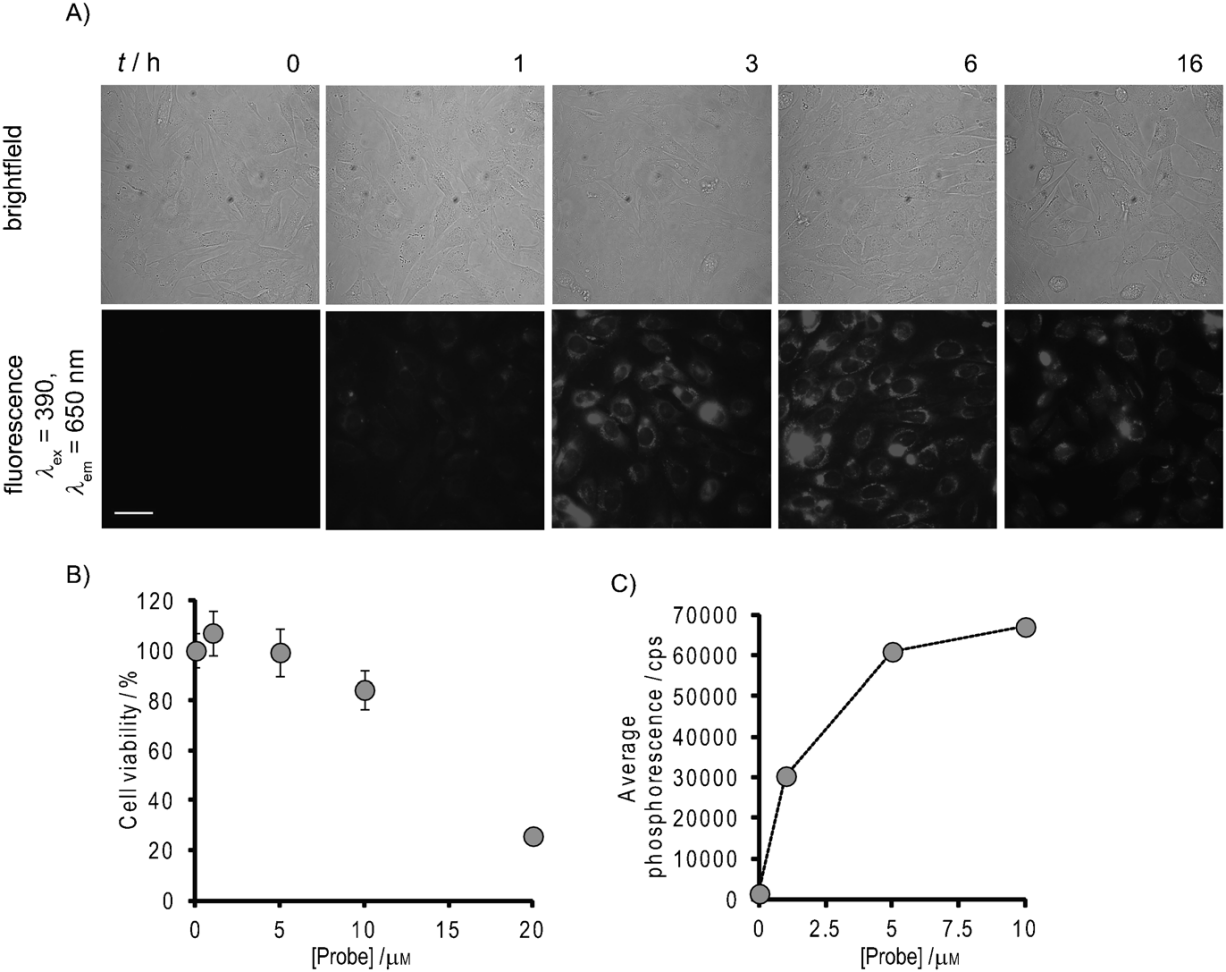
Time and concentration-dependence of cell staining and changes in cell viability for the Ir1 complex with MEF cells. A) Cells were incubated with the complex (10 μm) for the time indicated, washed, and analyzed by microscopy (brightfield and fluorescent images). Scale bars=50 μm. B) Changes in viability (total cellular ATP) measured after exposing the cells to Ir1 (0–20 μm) for 16 h. C) Average phosphorescence intensity signals from cells stained with Ir1 (0–10 μm).

To evaluate the cell entry mechanism as possible direct translocation, additional experiments were carried out. First, monitoring of kinetics of intracellular accumulation demonstrated that probe internalization was completed in about 3–6 h (see Ir1 data in Figure 3). This enables analysis of cell loading at low temperatures or upon ATP depletion. ATP depletion was induced by removing glucose from the medium and adding oligomycin (thus blocking both glycolysis and oxidative phosphorylation^2^). Under these conditions, we still saw efficient cell staining with Ir1 and Ir2, although cell appearance changed significantly (Figure S6). At low temperature (4 instead of 37 °C), when endocytosis occurs more slowly,^45^ we also observed faint intracellular staining with Ir1 and Ir2 (Figure S6). Based on these results, and considering that probe diffusion is also decreased at low temperature, we concluded that Ir1 and Ir2 utilize a direct translocation mechanism. Endocytosis-dependent cellular uptake is a commonly reported mechanism for oligoarginine and proline-rich peptide structures.^44^, ^46^–^48^ In this case, the internalized conjugate must undergo endosomal escape to reach the cytoplasm or other cellular organelles.^49^ We investigated cell loading in the presence of concanamycin A, an inhibitor of V-ATPase and lysosomal function, and observed a minor effect on Ir1 localization and a more profound effect on Ir2 (Figure S6). These data indicate that, even if these probes use endocytosis mechanism of cell entry, their escape from the endosomes is insignificant.

The Ir3 probe, which contains tumor-cell-targeting vector (RGD peptide), was tested for interaction with two cancer cell lines, HeLa and SH-SY5Y. After 1 h incubation with 5 μm of Ir3, we observed no detectable staining of the cells (Figure 2). This lack of binding functionality of the RGD sequences attached to the Ir–OEP moiety could be due to a number of factors, including steric factors, the positive charge on the adjacent Ir^III^ ion, or the hydrophobicity of the porphyrin core. On the other hand, the absence of cell staining by Ir3 confirms the essential role of cell-penetrating peptide moieties in intracellular delivery of the Ir1 and Ir2 probes.

### Application of Ir1 to intracellular O_2_ sensing

Efficient uptake of Ir1 and Ir2 by the cells allows for their use in sensing intracellular O_2_ (icO_2_). Photostability of these Ir–OEP-based probes is moderate and similar to that of PtCP conjugates. Unlike the highly photostable PtPFPP-based probes,^20^ the Ir–OEP probes are not very suitable for luminescence microscopy and FLIM applications but look promising for icO_2_ sensing experiments in RLD mode performed on TR-F readers in a similar manner as to other O_2_ probes.^17^, ^19^, ^50^, ^51^

**Figure 4:**
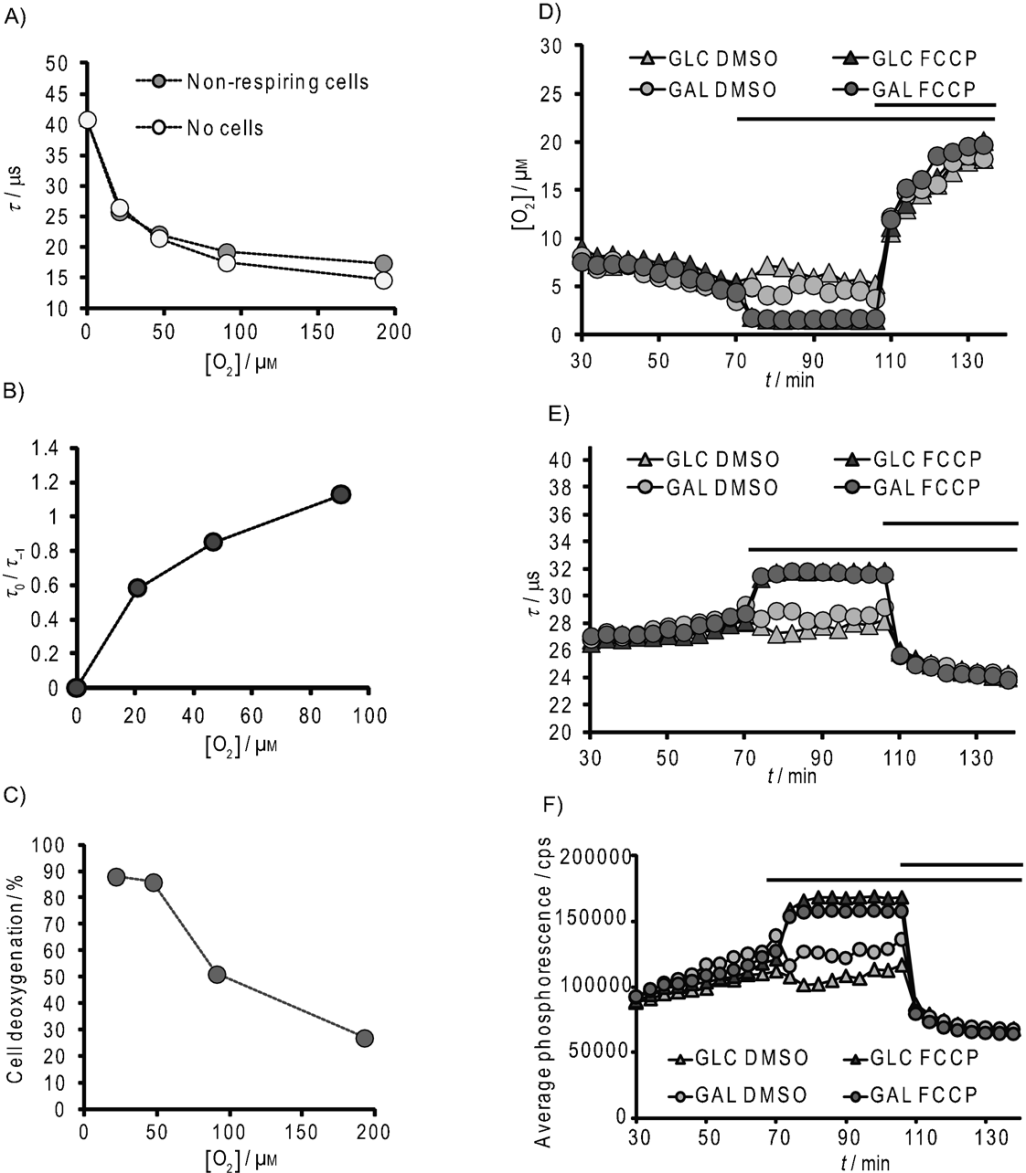
Sensing of icO_2_ in MEF cells with Ir1 probe. A) Phosphorescence lifetime (*τ*) calibrations in non-respiring MEF cells (probe in solution without cells is also shown; 21 % O_2_ corresponds to 200 μm dissolved O_2_). B) Stern–Volmer plot for Ir1 with non-respiring MEF cells. C) Relative deoxygenation of respiring cells in samples exposed to various levels of external pO_2_. D) Profiles of icO_2_ in MEF cells stained with Ir1 at rest and upon metabolic stimulation. E) and F) Raw phosphorescence lifetime (*τ*) and intensity respiration profiles of (D). The long bar shows the first treatment with FCCP or DMSO, the short bar shows the second treatment with AntA.

We first tested the Ir1 probe for possible toxic effects on MEF cells after 16 h of loading and found that at 20 μm its toxicity was high, but at 10 μm and below it was minimal (Figure 3 B). Figure 3 C shows that probe concentrations of 5–10 μm produce high phosphorescent signals with cells (50 000–60 000 cps at a delay time of 30 μs). The Ir1 probe was then calibrated with non-respiring MEF cells (at a staining concentration of 10 μm) with dissolved O_2_ ranging from 0 to 200 μm (Figure 4 C). The calibration data points (lifetimes ranging from ∼18–40 μs) were fitted with the following analytical function (*R*^2^=0.9865):


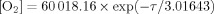
(1)

where [O_2_] is expressed in μm and *τ* in μs. The Stern–Volmer plots showing clear non-linearity (Figure 4 B) were fitted with the two-site model (see ref. 52 and Equation (2)), from which the following model parameters were determined: *F*=0.6; *K*_SV1_=0.074; *K*_SV2_=0.00027 (*r*^2^>0.999). Generally, *K*_SV2_ is significantly smaller than *K*_SV1_ (*K*_SV1_>*K*_SV2_×100); this indicates that one fraction of the probe is more easily accessible by oxygen than the other. Such behavior is not unusual for biological samples.^8^, ^53^


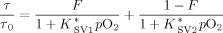
(2)

Relative deoxygenation of cells at different levels of external (atmospheric) hypoxia was calculated from calibration Equation (1) (Figure 4 C). One can see that when dissolved O_2_ is ≤50 μm, the cells are almost completely (>90 %) deoxygenated. Compared to the data obtained by Fercher et al. for the PtPFPP–RL100 probe (undefined intracellular location),^20^ our data show lower cell deoxygenation at similar concentrations of dissolved O_2_. This may be explained by the differences in intracellular localization between the Ir1 and PtPFPP–RL100 probes (i.e., PtPFPP is located closer to the mitochondria) and the possible existence of an intracellular O_2_ gradient, or by lower respiration activity in MEF cells stained with the Ir1 conjugate. Indeed, with moderate toxicity at 10 μm concentration, the oxidative phosphorylation activity can be compromised.

We also monitored O_2_ in MEF cells loaded with 5 μm of Ir1 in glucose(−)/galactose(+) medium exposed to hypoxic conditions (8 % ambient O_2_) and stimulated with 2 μm FCCP (uncoupler) and 10 μm antimycin A (ETC inhibitor) (Figure 4 D). In this case, basal O_2_ in resting cells of about 8–5 μm was reduced to ∼0.5 μm upon FCCP stimulation and increased to 20 μm upon AntA treatment (reoxygenation due to stopped respiration). The addition of DMSO (carrier) did not produce a significant response. Raw oxygenation profiles (in phosphorescence lifetime and intensity scales) corresponding to cellular respiration are shown in Figure 4 E and F.

## Conclusion

Overall, this study demonstrated that stable 1:2 complexes of Ir–porphyrins with peptides bearing histidine residues can be prepared by a simple and flexible ligand exchange procedure. With the examples of two cell-penetrating and one tumor-targeting peptides, we showed the flexibility of this methodology, which can be extended to other biologically relevant structures. The resulting complexes displayed good solubility in aqueous media, showing bright phosphorescence and unquenched lifetimes above 40 μs. The Ir1 and Ir2 probes showed cell-penetration ability that involves a direct translocation mechanism, broad cell specificity, and efficient staining of different cell lines. Their intracellular distribution was close to the endoplasmic reticulum. Such probes represent useful tools for O_2_ sensing and particularly for real-time monitoring of icO_2_, which can be realized on existing commercial TR-F readers or, with additional modifications, in ratiometric intensity-based detection formats. However, moderate photostability limits their use in O_2_ imaging.

## Experimental Section

**Materials:** Luminescent cell viability kit CellTiter-Glo was from Promega (Madison, WI, USA), fluorescent probe ER Tracker Green was from Invitrogen (Bio Sciences, Dun Laoghaire, Ireland). Standard 96-well cell culture and white 96-well plates (for CellTiter Glo Kit) were purchased from Greiner Bio-One (Frickenhausen, Germany). Glass-bottom multi-well inserts were from Ibidi (Martinsried, Germany). All other reagents were from Sigma–Aldrich Ltd. (Dublin, Ireland). Peptides with C-terminal amidation and confirmed structure (MS) and purity (HPLC) were from GenScript (Piscataway, NJ, USA).

**Synthesis and characterization of conjugates Ir1, Ir2 and Ir3:** Ir–OEP–CO-Cl (2–3 μmol, produced as described in refs. 39, 54) and the peptide (4–5 equiv) were added to a screw-cap glass vial, dissolved in 2-ethoxyethanol (2 mL), incubated at 75 °C for 1 h, and then left to react overnight at 60 °C. After the absorption band corresponding to Ir–OEP–CO-Cl at 550 nm was no longer visible, the reaction was stopped and the solvent removed. Next, water (2 mL) was added to the dry red residue, and the solution was sonicated for 15 min, followed by removal of the insoluble fraction by centrifugation. The water-soluble fraction was purified by HPLC (Agilent 1100 Series) on a semi-preparative column VP 250/10 Nucleodur 100–5 RP-C18 using a 0.1 % aqueous acetic acid/MeOH gradient. Typical product yield after purification was 30±5 %. ^1^H NMR spectra were recorded in D_2_O on a 300 MHz spectrometer from Brucker. Mass spectrometric analysis was carried out on a triple quadrupole API 2000 mass spectrometer (Applied Biosystems/Sciex, Concord, Canada) equipped with a positive electrospray ionization (ESI) interface under full-scan mode (200–1800 amu).

**Photophysics and phosphorescence lifetime measurements:** Absorption spectra were recorded on an 8453 UV/Vis diode-array spectrophotometer (Agilent), and luminescence spectra were collected on a LS50B spectrometer (PerkinElmer). Absolute quantum yields of emission were measured on a Horiba FluoroLog3 (http://www.horiba.com) equipped with a Quanta-phi integrating sphere. Quantum yields in PBS containing 10 % fetal bovine serum were measured under oxygen-free conditions as described in ref. 17.

Phosphorescence lifetimes were assessed on a Cary Eclipse fluorescence spectrometer (Varian-Agilent) with 380 nm excitation, 650 emission, and 30 μs delay time. For rapid lifetime determination on a Victor 2 reader (PerkinElmer), the “time-resolved fluorescence” mode was used, with D340 excitation and D642 emission filters, measuring at two delay times (*t*_1_=30 μs and *t*_2_=40 μs) with a gate time of 100 μs and a total counting cycle of 1 s. Phosphorescence lifetime (τ) was calculated according to Equation (3)


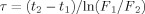
(3)

where *F*_1_ and *F*_2_ correspond to TR-F readings at delay times *t*_1_ and *t*_2_.

**Cell culture:** Mouse embryonic fibroblasts (MEFs), African green monkey kidney (COS-7), human epithelial carcinoma (HeLa), human neuroblastoma (SH-SY5Y), and rat pheochromocytoma (PC12) cells from ATCC (Manassas, VA, USA) were cultured as described previously, using DMEM supplemented with 10 %FBS (for MEF, COS-7, HeLa, and SH-SY5Y cells) or RPMI1640 supplemented with horse serum and FBS (for PC12 cells) media and collagen-poly-D-lysine coated glass bottom minidishes (for microscopy analysis) or collagen IV-coated 96-well plates (for plate reader measurements). Primary neurons from rat brain were kindly provided by Dr. Y. Nolan (Anatomy Department, UCC). Cell viability was assessed by measuring total cellular ATP with a CellTiter-Glo luminescent kit (Promega), according to manufacturer's recommendations.

Live cell microscopy was performed on a fluorescent microscope Axiovert 200 (Carl Zeiss, Goettingen, Germany) equipped with an LED excitation module (LaVision GmbH, Goettingen, Germany). An UV LED (390 nm) and PtCP filter cube (*λ*_ex_=390/40 nm, *λ*_em_=655/40 nm) were used for imaging the Ir–porphyrins. Cells were incubated with Ir1 and Ir2, typically for 6–16 h, then washed three times, counterstained with ER Tracker Green (1 μm, 30 min), washed again, and imaged. For ATP depletion experiments, cells were incubated in glucose-free DMEM supplemented with 10 % FBS, galactose (10 mm), sodium pyruvate (1 mm), and HEPES (20 mm), pH 7.2 for 1.5 h, then with oligomycin (10 μm) for 0.5 h, followed by staining with Ir1 and Ir2 (10 μm concentration) for 6 h in this medium and fluorescence microscopy imaging.

Phosphorescence lifetime measurements were conducted on a Victor 2 plate reader as described above. For probe calibration and monitoring of oxygenation under graded hypoxia, cells stained with Ir1 (10 μm, 16 h) were exposed to different levels of atmospheric O_2_ in a glove box (Coy Scientific), with 60 min pre-incubation and 30–60 min measurement at each O_2_ concentration in the presence of antimycin A (10 μm). To achieve 0 % O_2_, a solution containing glucose (100 mm) and glucose oxidase (50 μg mL^−1^, Sigma G7141) was added to the cells (1/10 of the volume) exposed to 1–2 % O_2_. For stimulation experiments, 10× stock solutions of effectors were added during measurement to produce the indicated final concentrations. Relative cell oxygenation was calculated as described previously^55^ using phosphorescence lifetime data obtained with respiring and non-respiring (antimycin A-treated) cells under the same ambient O_2_ concentrations.

**Data assessment:** The results of plate reader experiments were processed in Microsoft Excel and Origin 6.0 for fitting the calibration. The data represent average values with standard deviations shown as error bars. To ensure consistency, all experiments were performed in triplicate.

## Supplementary material

Detailed facts of importance to specialist readers are published as ”Supporting Information”. Such documents are peer-reviewed, but not copy-edited or typeset. They are made available as submitted by the authors.
