# Supplementary material for: Complexes of IrIII-Octaethylporphyrin with Peptides as Probes for Sensing Cellular O2
Source: Chembiochem. 2012 Apr 24;13(8):1184–90. doi: 10.1002/cbic.201200083 (PMC3437475; doi:10.1002/cbic.201200083)
Supplement: Supplementary file 1 [file cbic0013-1184-SD1.pdf]

CHEMBIOCHEM

Supporting Information

© Copyright Wiley-VCH Verlag GmbH & Co. KGaA, 69451 Weinheim, 2012

Complexes of Ir^{III}-Octaethylporphyrin with Peptides as Probes for Sensing Cellular O_2

Klaus Koren,[a] Ruslan I. Dmitriev,[b] Sergey M. Borisov,[a] Dmitri B. Papkovsky,*[b] and Ingo Klimant[a]

cbic_201200083_sm_miscellaneous_information.pdf

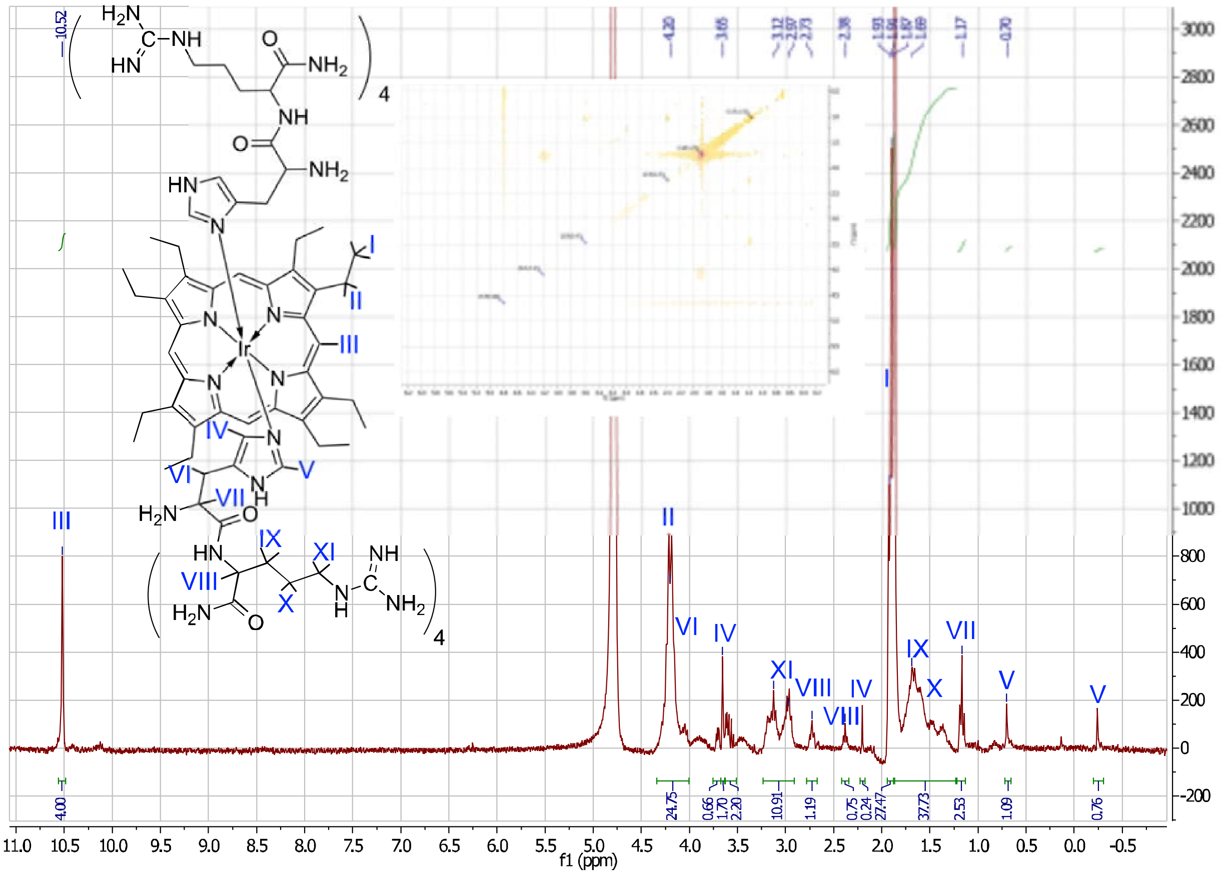

Fig. S1. ^{1}H NMR spectrum of Ir1.

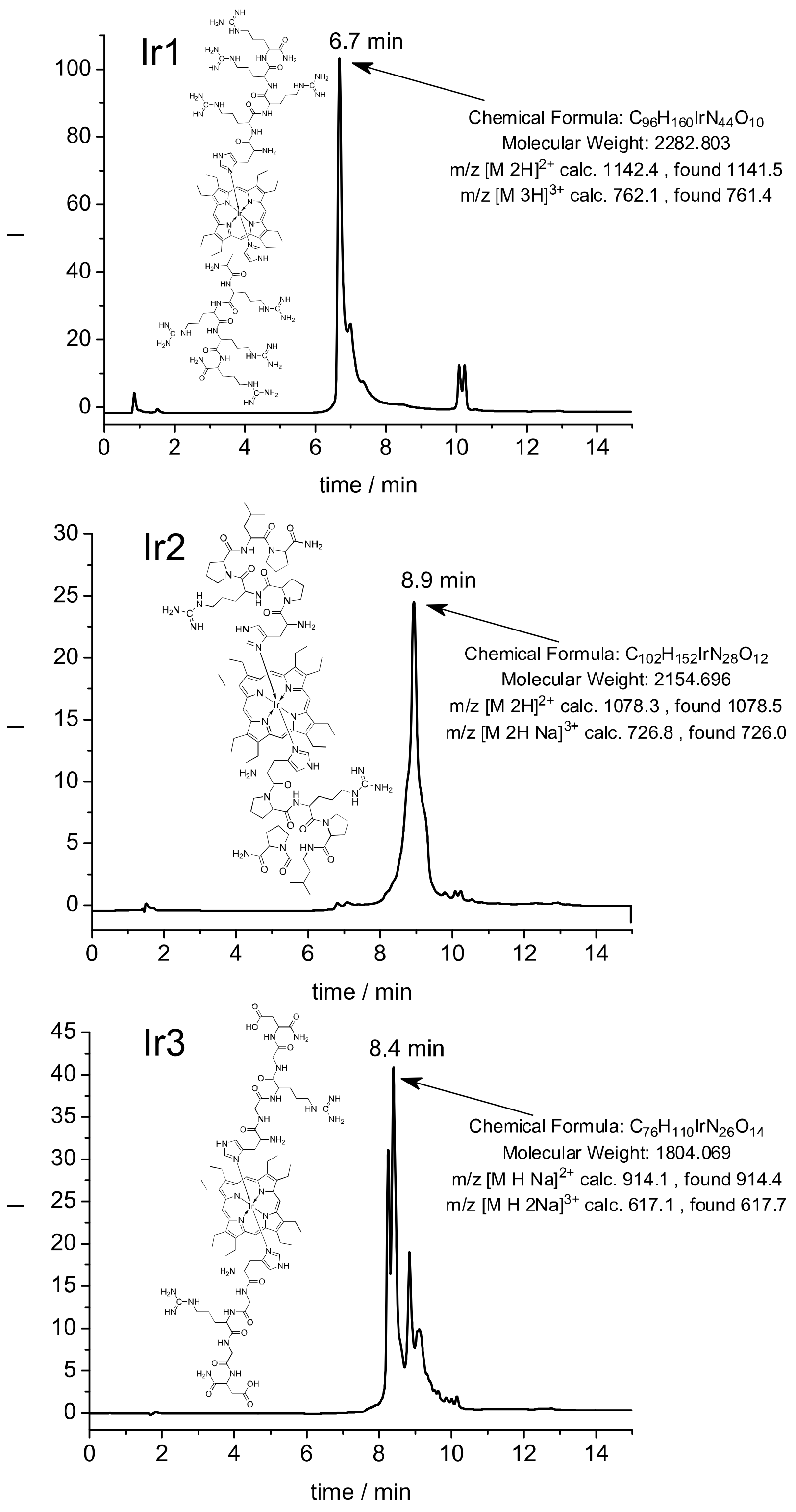

Fig. S2. RP-HPLC chromatograms of Ir1, Ir2 and Ir3, with structural M/S data evaluated on a triple quadrupole spectrometer.

A: Cary Eclypse results. 0% O_2 in solution

Equation: A1 exp(-time/TAU1) + C

Ir1

TAU1 (ms)	A1	C	S.D.
0.058±0.0	663.6921±48.8	2.0116±2.2	4.0460

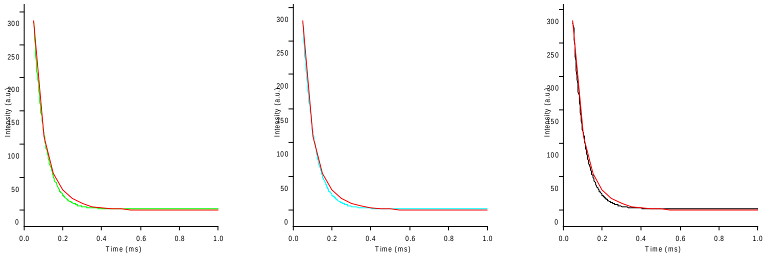

Ir2

TAU1 (ms)	A1	C	S.D.
0.069±0.0	1149.2780±39.6	2.5998±2.5	4.4624

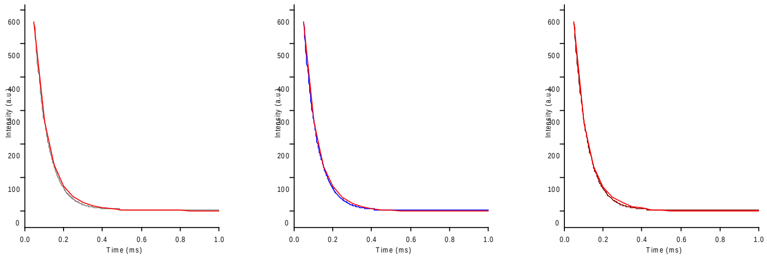

Ir3

TAU1 (ms)	A1	C	S.D.
0.047±0.0	1862.7120±140.0	3.5822±4.1	7.7163

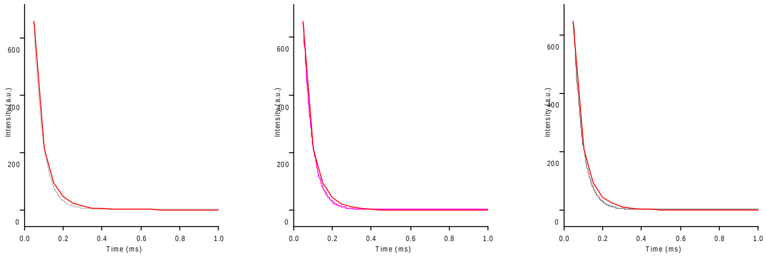

B:

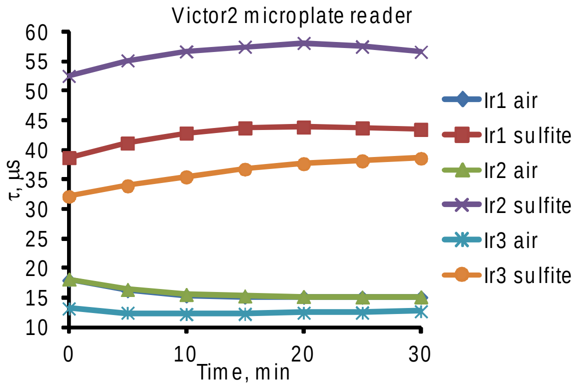

Fig. S3. Phosphorescence decay curves and lifetime values for the Ir conjugates in aqueous solutions (PBS).

Table S4.

Phosphorescence intensities (F1, t = 30 μs) for various concentrations of conjugate Ir1 obtained in PBS/1% FBS on Victor2 microplate reader.

	Air		Sulfite (deoxygenated)	
Ir1 μM	average	stdev	average	stdev
10	151445.0	49388.15	3316721	770843.6
1	67389.6	22185.56	483681	75986.63
0.1	16504.3	4254.608	52404.33	9896.208
0.01	1985.3	1012.473	9293.667	9091.802
0.001	626.2	92.65363	841.8889	30.21359
0	407.7	43.48563	418.7778	17.03509

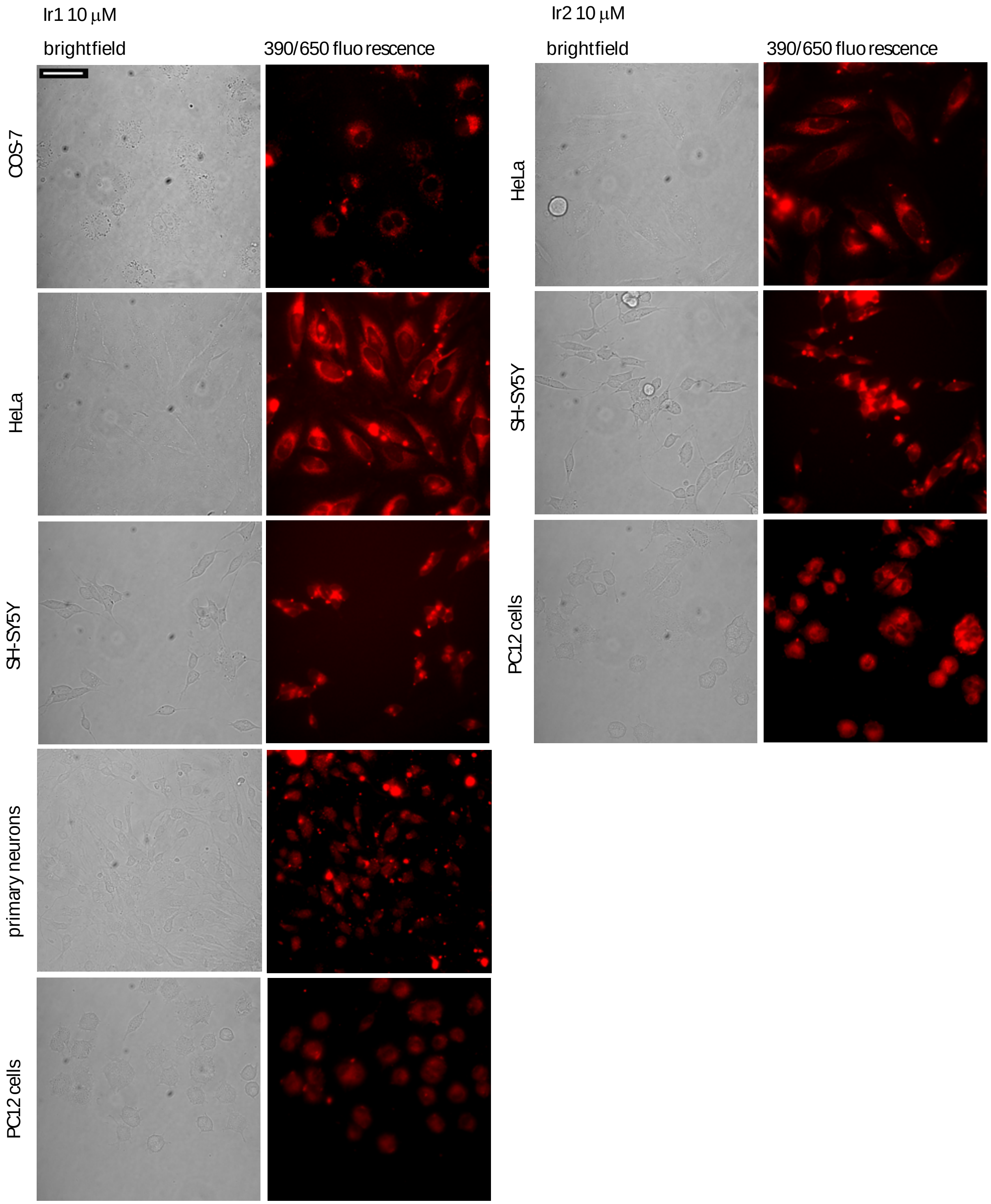

Fig. S5. Brightfield and fluorescent images of other cell lines stained with Ir1 and Ir2 probes. Scale bar 50 μm.

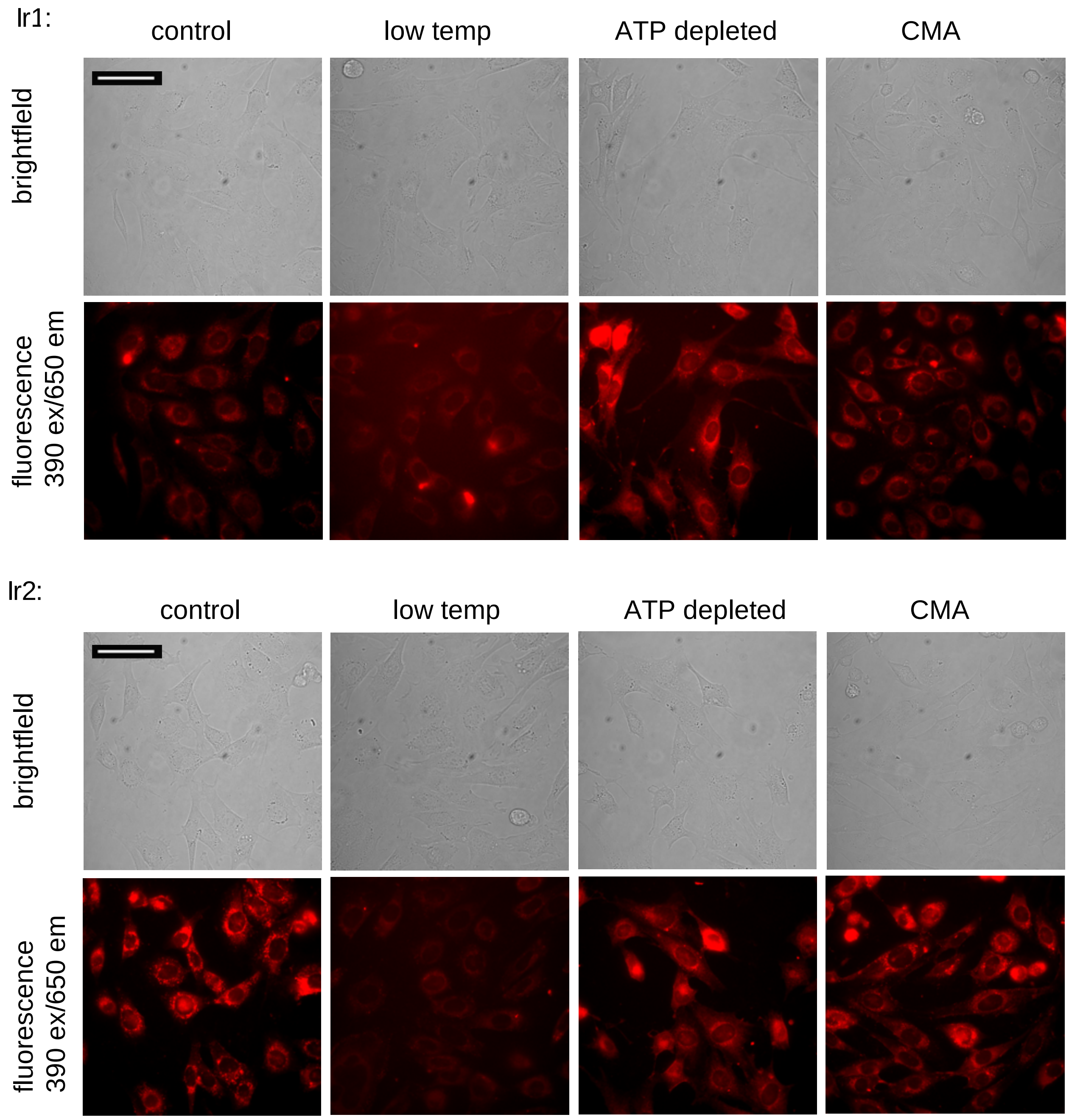

Fig. S6. Effects of temperature, ATP depletion and inhibition of lysosomal function on intracellular accumulation of the probes in MEF cells. Ir1 and Ir2 were loaded (at 10 μM for 6 h) in cells exposed to ATP depletion, concanamycin A (CMA) treatment or low temperature (4 °C), then washed and investigated by fluorescence microscopy (brightfield and fluorescence images). Scale bar 50 μm.
